# Boldine Attenuates Synaptic Failure and Mitochondrial Deregulation in Cellular Models of Alzheimer’s Disease

**DOI:** 10.3389/fnins.2021.617821

**Published:** 2021-02-19

**Authors:** Juan P. Toledo, Eduardo J. Fernández-Pérez, Ildete L. Ferreira, Daniela Marinho, Nicolas O. Riffo-Lepe, Benjamin N. Pineda-Cuevas, Luis F. Pinochet-Pino, Carlos F. Burgos, A. Cristina Rego, Luis G. Aguayo

**Affiliations:** ^1^Laboratory of Neurophysiology, Department of Physiology, Universidad de Concepción, Barrio Universitario, Concepción, Chile; ^2^CNC-Center for Neuroscience and Cell Biology, University of Coimbra, Coimbra, Portugal; ^3^IIIUC-Institute for Interdisciplinary Research, University of Coimbra, Coimbra, Portugal; ^4^FMUC-Faculty of Medicine, University of Coimbra, Coimbra, Portugal

**Keywords:** Alzheimer’s disease, Boldine, mitochondria, synaptic transmission, intracellular Ca^2+^

## Abstract

Alzheimer’s disease (AD) is the most common cause of senile dementia worldwide, characterized by both cognitive and behavioral deficits. Amyloid beta peptide (Aβ) oligomers (AβO) have been found to be responsible for several pathological mechanisms during the development of AD, including altered cellular homeostasis and synaptic function, inevitably leading to cell death. Such AβO deleterious effects provide a way for identifying new molecules with potential anti-AD properties. Available treatments minimally improve AD symptoms and do not extensively target intracellular pathways affected by AβO. Naturally-derived compounds have been proposed as potential modifiers of Aβ-induced neurodysfunction and cytotoxicity based on their availability and chemical diversity. Thus, the aim of this study was to evaluate boldine, an alkaloid derived from the bark and leaves of the Chilean tree *Peumus boldus*, and its capacity to block some dysfunctional processes caused by AβO. We examined the protective effect of boldine (1–10 μM) in primary hippocampal neurons and HT22 hippocampal-derived cell line treated with AβO (24–48 h). We found that boldine interacts with Aβ *in silico* affecting its aggregation and protecting hippocampal neurons from synaptic failure induced by AβO. Boldine also normalized changes in intracellular Ca^2+^ levels associated to mitochondria or endoplasmic reticulum in HT22 cells treated with AβO. In addition, boldine completely rescued the decrease in mitochondrial membrane potential (ΔΨm) and the increase in mitochondrial reactive oxygen species, and attenuated AβO-induced decrease in mitochondrial respiration in HT22 hippocampal cells. We conclude that boldine provides neuroprotection in AD models by both direct interactions with Aβ and by preventing oxidative stress and mitochondrial dysfunction. Additional studies are required to evaluate the effect of boldine on cognitive and behavioral deficits induced by Aβ *in vivo*.

## Introduction

Alzheimer’s Disease (AD) is the most prevalent neurodegenerative disorder characterized by cognitive and behavioral deficits (Terry et al., 1991) that is expected to reach 82 million cases in 2030 (Weidner and Barbarino, 2019). Age-related forms of dementia lead to sporadic AD. Conversely, less than 1% of cases are associated with familial forms due to mutations in the presenilin 1 or presenilin 2 genes involved in the processing of the amyloid precursor protein (APP) (Masters et al., 2015). AD pathology shows progressive neuronal damage and atrophy in vulnerable brain regions and circuits involved in memory, specifically in the hippocampus and cerebral cortex. These events appear to be preceded by synaptic and neuronal dysfunction. Neuropathologically, AD is characterized by the presence of extracellular plaques mainly composed of misfolded fibrillar amyloid beta peptide (Aβ) derived from the amyloidogenic processing of the APP by β- and γ-secretases, and intraneuronal neurofibrillary tangles consisting of aggregates of hyperphosphorylated tau protein (Ferreira et al., 2012b; Cline et al., 2018, for review). We previously reported that treatment (24 h) of hippocampal neurons with Aβ oligomers (AβO), but not monomers or fibers, reduced synaptic transmission evidenced by a large reduction in frequency of evoked and miniature currents, together with a number of presynaptic markers, that included SV2 (Parodi et al., 2010). In more recent studies, we have confirmed these original findings on the synaptoxicity of AβO and further characterized the toxic components of these Aβ assemblies by oligomerization time curves, characterization of dimer and tretramers and by atomic force microscopy that reported nanometric structures (González-Sanmiguel et al., 2020).

Additionally, synaptic depression was associated with disruption of glutamatergic transmission that results from internalization of *N*-methyl-D-aspartate (NMDA) receptors (NMDARs) caused by AβO, ultimately leading to early cognitive deficits (Snyder et al., 2005; Cline et al., 2018, for review). Accordingly, it was previously demonstrated that AβO (1 μM) pre-exposure reduces NMDA-evoked intracellular free Ca^2+^ (Ca^2+^_*i*_). Additionally, AβO *per se* induces an immediate Ca^2+^_*i*_ rise involving GluN2B-containing NMDARs in cortical neurons (Ferreira et al., 2012a).

On the other hand, mitochondria play a pivotal role in energy metabolism and neuronal Ca^2+^ buffering in neurons. Indeed, mitochondria take up Ca^2+^ in response to small increases in intracellular Ca^2+^_*i*_ levels (Naia et al., 2017, for review). In addition, mitochondria are in close physical contact with the endoplasmic reticulum (ER) in a structure formed by a myriad of proteins and several tethers named mitochondria-associated membranes (MAMs). This tight juxtaposition favors Ca^2+^ exchange and signaling between the two organelles (Csordás et al., 2018). In our previous studies, we found that neuron exposure to AβO plus NMDA (mimicking imbalanced glutamatergic neurotransmission) potentiates the increase in Ca^2+^_*i*_ levels compared to AβO or NMDA alone (Ferreira et al., 2012a), leading to augmented mitochondrial Ca^2+^ retention and mitochondrial depolarization through a pathway that involves ER IP3R and the mitochondrial Ca^2+^ uniporter (MCU) (Ferreira et al., 2015), highlighting the involvement of ER-mitochondria interplay in abnormal Ca^2+^ homeostasis. In addition, AβO depletes ER-Ca^2+^ through IP3R- and RyR-mediated Ca^2+^ release increasing Ca^2+^_*i*_ levels and therefore compromising cell survival (Resende et al., 2008). In addition, generation of Aβ-induced ion channels cause aberrant cell excitability and accumulation of Ca^2+^_*i*_ (Snyder et al., 2005; Lei et al., 2016) causing an enhanced release of neurotransmitters and synaptic depletion (Sepúlveda et al., 2014). Ultimately, the sum of all of these conditions causes a loss of synaptic plasticity and cell death (Lambert et al., 1998; De Felice et al., 2008). Mitochondrial Ca^2+^ overload induces disruption of mitochondrial respiration and ATP synthesis. Concomitantly, excessive production of reactive oxygen species (ROS) triggered by mitochondrial dysfunction and ER stress culminates in the oxidative damage observed in AD cells and animal models, as well as in AD patient’s biological fluids and peripheral blood mononuclear cells (PBMCs) (Ferreira et al., 2012b; Mota et al., 2015).

Cholinesterase inhibitors and memantine (uncompetitive NMDAR antagonist) are the only drugs approved by the Food and Drug Administration (FDA) to slowdown the cognitive symptoms in the early and late stages of AD, respectively, however, neither of them inhibit the disease progression. Therefore, the development and testing of new drugs, such as natural compounds, is imperative as a primary prevention of cognitive decline associated with AD (Tewari et al., 2018; Cummings et al., 2019).

Boldine is an aporphinoid alkaloid derived from the wood and leaves of the Chilean tree *Peumus boldus* (commonly known as boldo) and traditionally consumed in South America (Cassels et al., 2018). Boldine structure is very similar to apomorphine, a molecule that was previously demonstrated to be neuroprotective in a 3xTg-AD mouse model by promoting the degradation of intraneuronal Aβ (Himeno et al., 2011). Boldine has been proposed to have potent antioxidant (Bannach et al., 1996), anti-inflammatory (Backhouse et al., 1994), anticonvulsive (Moezi et al., 2018), antihypertensive (Lau et al., 2013) and antidiabetic properties (Hernández-Salinas et al., 2013). In a previous study, a reduction in the inflammatory brain response was reported in glial cells in an APPswe/PS1dE9 model of AD (Yi et al., 2017), although these investigators used much higher concentrations for *in vitro* experiments than those used in the present study.

Therefore, the aim of this study was to characterize the effects of boldine on AβO aggregation *in vitro* on AβO-induced synaptic impairment and postsynaptic currents in mature hippocampal neurons and on mitochondrial and ER–Ca^2+^ retention, mitochondrial function, and ROS levels in a HT22 mouse hippocampal-derived cell line. Overall, our data with boldine shows that it attenuates Aβ-induced synaptic deficits and mitochondrial dysfunction in AD cell models.

## Materials and Methods

### Reagents

Boldine was purchased from Sigma (Germany) and a 50 mM stock solution was prepared in DMSO and stored at −20°C according to manufacturer indications. Synthetic Aβ_1__–__4__2_ was purchased from Bachem (Bubendorf, Switzerland) and reconstituted following the instructions of the manufacturer. Aβ_1__–__4__2_ oligomers used in HT22 cells (referred as AβO) were prepared as previously described (Ferreira et al., 2012a). For electrophysiological studies, AβO were prepared as previously reported (Peters et al., 2013; González-Sanmiguel et al., 2020). For instance, Aβ_1__–__4__2_ was dissolved in hexafluoroisopropanol (HFIP,10 mg/mL) and stored in aliquots at −20°C. For AβO preparation (80 μM), aliquots of 5 mL were added to 137.5 mL ultrapure water in an Eppendorf tube. After 15 min incubation at room temperature, the samples were centrifuged at 14,000 g for another 15 min, and the supernatant fraction transferred to a new tube. The samples were stirred at 500 rpm using a Teflon-coated microstir bar for 24–48 h at room temperature (approximately 22°C) and stored at 4°C until required. Fura2-AM and TMRM^+^ were purchased from Molecular Probes-Invitrogen (Eugene, OR, United States). Poly-L-lysine, MitoPY1, oligomycin, FCCP and 3-(4,5-dimethyl-2-thiazolyl)-2,5-diphenyl-2H-tetrazolium bromide (MTT) were purchased from Sigma Chemical Co. (St. Louis, MO, United States). Thapsigargin was purchased from Tocris Bioscience (Bristol, United Kingdom). The monoclonal mouse anti-synaptic vesicle protein 2 (SV2) antibody was from Developmental Studies (HybridomaBank, Iowa City, IA, United States) and rabbit anti-MAP2 was from Santa Cruz Biotechnology (Dallas, TX CA, United States). Anti-rabbit IgG conjugated with Cy5 and anti-mouse IgG conjugated with Cy3 were from Jackson Immuno Research Laboratories (West Grove, PA, United States). DAKO mounting medium was purchased from Agilent, United States. All other reagents were of analytical grade. The final concentration of DMSO in assays was not higher than 1% v/v.

### Docking Simulations

In order to study the *in silico* interaction between boldine and Aβ_1__–__4__2_, a protein-ligand docking was performed. First, the interaction grids (15 Å) for the C-terminal, central regions and full peptide were generated using the structure of monomeric Aβ_1__–__4__2_ (PDB ID: 1IYT) with Glide (Schrödinger, LLC, New York, NY). Boldine structure was obtained from PubChem (CID: 10154). A protein-ligand docking was performed using Glide with a high precision (XP) configuration. The complexes were analyzed using the docking score provided by Glide and the calculation of the MM-GBSA ΔG_*bind*_ using Prime (Schrödinger, LLC, New York, NY). All images presented were created with PyMOL (Schrödinger, LLC, New York, NY).

### Aggregation Assay

For the aggregation and experiments in hippocampal neurons, we used a synthetic Aβ_*1–42*_ peptide obtained from Genemed Syn-thesis, Inc. (United States). Oligomeric species were prepared by measuring absorbance of Aβ samples at 23°C (Peters et al., 2020). We studied the inhibition of Aβ_*1–42*_ (40 μM) aggregation with GBP for 24 h under a stirring condition of 500 rpm. Absorbance at 405 nm was measured for 9 h with readings every 20 min using a Novostar microplate reader (BMG Labtech, United States). Protein absorbance was measured at 482 nm and all the experiments were performed in triplicates. Briefly, 40 μM Aβ was added to a 96 well plate in the presence and absence of 10 and 100 μM boldine.

### Primary Culture of Hippocampal Neurons

Hippocampal neurons were obtained from C57BL/J6 mice or Sprague-100 Dawley rat embryos as previously described (e.g., Aguayo and Pancetti, 1994). Animal care and protocols were in accordance with the National Institutes of Health (NIH) recommendations and approved by the Ethics Committee at the University of Concepcion. Briefly, hippocampal tissue was harvested from 18 to 19 days old embryos. The pregnant animal was anesthetized with CO_2_ and subsequently euthanized by cervical dislocation. The embryos were removed and rapidly decapitated. Brains were removed and the hippocampi were dissected from the cortices free of meninges. The hippocampus was mechanically and enzymatically dissociated (Aguayo and Pancetti, 1994). After being isolated, neurons were platted at 250,000 cells/mL on coverslips precoated with poly-L-lysine in 90% minimal essential medium (MEM; Gibco, Grand Island, NY, United States), 5% heat-inactivated horse serum (Gibco), 5% fetal bovine serum (Gibco) and supplemented with N3 (mg/mL: BSA 1, putrescine 3.2, insulin 1, apotransferrin 5, corticosterone 0.5 (g/mL: sodium selenite 5, TH3 0.5, progesterone 0.6). Cultures were maintained in a humidified atmosphere at 37°C containing 95% air and 5% CO_2_ for 8–10 days. Culture medium was partially replaced every 3 days. Hippocampal neurons (10 DIV) were treated with 1 μM AβO in the presence or absence of 10 μM boldine for 24 h.

### Electrophysiological Recordings

For voltage-clamp experiments in the whole cell mode, culture medium was replaced with a normal external solution (NES) containing: 150 mM NaCl, 5.4 mM KCl, 2 mM CaCl_2_, 1 mM MgCl_2_, 10 mM glucose and 10 mM HEPES, pH 7.4/NaOH; 320 mOsm/L). Cells were stabilized at room temperature for 20 min before experiments. The internal solution used to record spontaneous post-synaptic currents (sPSCs) contained 120 mM KCl, 2.0 mM MgCl_2_, 2 mM Na_2_ATP, 10 mM BAPTA, 0.5 mM NaGTP and 10 mM HEPES, pH 7.4/KOH; 310 mOsm/L). All currents were recorded by adjusting the membrane potential to −60 mV using an Axopatch-200B amplifier (Molecular Devices, United States) and an inverted microscope (Nikon Eclipse TE200-U, Japan). The acquisition was made using a computer connected to the recording system with a Digidata 1440A acquisition card (Molecular Devices, United States) and the pClamp10 software (Molecular Devices, United States). Electrodes with a resistance of 4–5 MΩ were pulled from borosilicate capillaries (WPI, United States) in a horizontal puller (P1000, Sutter Instruments, United States). A 5 mV pulse was used to monitor series resistance throughout the recording period and only cells with a stable access resistance (less than 15 MΩ and that did not change more than 20%) were included for data analysis. After acquiring the synaptic recording, the area under the current trace was integrated (pA⋅ms) and expressed as charge transferred (nC) during the whole recording period (2 min) using Clampfit 10.5 (Molecular Devices, United States).

### Immunocytochemistry

Hippocampal neurons were fixed for 15 min with 4% paraformaldehyde in PBS. Thereafter, cells were incubated with permeabilization and blocking solution with 0.1% Triton X-100 in horse serum (HS):PBS 1:10 for 20 min. Cells were then incubated with primary antibodies [monoclonal mouse anti-synaptic vesicle 2 protein (SV2) antibody (1:200) and rabbit anti-MAP2 (1:300)] overnight at 4°C followed by incubation with secondary antibodies [anti-rabbit IgG conjugated with Cy5 (1:500) and anti-mouse IgG conjugated with Cy3 (1:500)] for 2 h at room temperature. All antibodies were diluted with HS (10%) in PBS. Coverslips were mounted in DAKO mounting medium and cells observed under a spectral confocal laser scanning microscope (LSM780, Zeiss, Germany) using a 63 × 1.4 numerical aperture oil immersion objective (Zeiss, Germany). Images of 16-bit were collected using a pixel time of 1.58 μs and a pixel size of 110 nm.

### Quantification of SV2 Fluorescent Puncta

In fluorescence microscopy, light undergoes diffraction while traveling in an imaging system leading to image blurring and limiting visual access to details. The blurring is characterized by a point-spread function (PSF) that along with the original image can be used in a deconvolution algorithm to restore microscopic details. Therefore, using the Richardson−Lucy algorithm provided by DeconvolutionLab2 plugin (Sage et al., 2017) in Image J (NIH) and a theoretical PSF (based on imaging parameters), we deconvolved and analyzed the confocal micrographs of neurons that were immunostained for synaptic vesicle 2 (SV2), a presynaptic marker. Deconvolution was followed by maximum intensity z-projection and background adjustment. Using the MAP-2 signal, we generated a mask to only obtain the signal of SV2 in the neuron where the analysis was carried out. The micrographs were used to quantify the size and number of fluorescent punctas of SV2 (or clusters) on the first 20 μm of neuronal primary processes using measuring tools from Image J software. At least 30 processes *per* condition were counted.

### HT22 Cell Culture and Treatment

HT22 cells, a mouse hippocampal cell line obtained from the immortalization of primary hippocampal neurons using a temperature sensitive SV40 T-antigen and subcloned from HT4 cells based on sensitivity to glutamate (Davis and Maher, 1994), were obtained from Dr. Dave Schubert (Salk Institute, La Jolla). The cells were grown in 75 cm^2^ culture flasks in high-glucose Dulbecco’s Modified Eagle’s medium (DMEM) supplemented with 10% heat inactivated (HI) fetal bovine serum (FBS), 12 mM NaHCO_3_, 5 mM HEPES and 100 μg/mL penicillin-streptomycin, pH 7.3 in a humidified incubator with 5% CO_2_ and 95% air at 37°C. When 80% confluency was reached, cells were detached using a Ca^2+^-Mg^2+^-free dissociation medium containing 140 mM NaCl, 8.1 mM Na_2_HPO_4_, 1.47 mM KH_2_PO_4_, 1.47 mM KCl, 0.55 mM EDTA, pH 7.3 and then centrifuged at 800 rpm for 5 min and subsequently sub-cultured or plated at a density of 0.005 × 10^6^ cell/well in 96-multiwell plates. Twenty four hour after plating, cells were treated with 1 μM AβO in the presence or absence of 1, 10 or 100 μM boldine in culture conditioned medium (medium where the cells were plated) for 24 h at 37°C in a humidified culture chamber containing 95% O_2_ and 5% CO_2_. The effect of boldine alone was also tested.

### MTT Assay

Cell viability was determined using the colorimetric MTT assay based on the reduction of MTT into an insoluble formazan product via nicotinamide adenine dinucleotide phosphate (NADH)-dependent dehydrogenases by viable cells. HT22 cells were incubated with 0.5 g/L MTT in Krebs medium containing 135 mM NaCl, 5 mM KCl, 1.8 mM CaCl_2_, 0.4 mM KH_2_PO_4_, 1 mM MgSO_4_, 5.5 mM glucose, 20 mM HEPES, pH 7.4 for 2 h at 37°C in the dark. Krebs medium was removed and formazan crystals were dissolved in 0.04 M HCl in isopropanol. The absorbance at 570 nm was then measured spectrophotometrically using a SpectraMax plus microplate reader (Molecular Devices, United States).

### Mitochondrial and ER-Ca^2+^ and ΔΨm Measurement

For mitochondrial-Ca^2+^ and ΔΨm measurements, HT22 cells were rinsed with Krebs medium and co-loaded with the high affinity Ca^2+^ probe Fura2-AM (5 μM) that accumulates in the cytoplasm plus the ΔΨm sensing dye TMRM^+^ (300 nM; under quenched mode) that accumulates in polarized mitochondria, for 40 min at 37°C. Cells were then rinsed with Krebs medium and fluorescence was measured in the presence of TMRM^+^ (in order to prevent leakage) using a Spectrofluorometer Gemini EM (Molecular Devices, United States). The fluorescence was recorded for both Fura2 at 340/380 nm excitation and 510 emission, and for TMRM^+^ at 540 nm excitation and 590 nm emission. After the establishment of a 2-min baseline, cells were stimulated with 2 μM FCCP to induce maximal mitochondrial depolarization in the presence of 2 μg/mL oligomycin, to prevent ATP hydrolysis. The cytoplasmic Ca^2+^ increase upon mitochondrial Ca^2+^ release was recorded as an increase in the Fura2 fluorescence, whereas ΔΨm was evaluated by the increase in TMRM^+^ fluorescence signal in response to mitochondrial full depolarization. For ER-Ca^2+^ measurements, cells were incubated in Fura2-containing Krebs medium for 30 min at 37°C. Following a washing step, fluorescence was measured in Ca^2+^-free Krebs medium. After the baseline establishment, cells were stimulated with 1 μM thapsigargin, a selective non-competitive inhibitor of sarcoendoplasmic reticulum Ca^2+^-ATPase (SERCA), that leads to ER-Ca^2+^ depletion. The increase in Fura2 fluorescence signal was recorded and analyzed as relative levels of ER-Ca^2+^. Fluorescence was measured using a Spectrofluorometer Gemini EM (Molecular Devices, United States). Ca^2+^ levels and ΔΨm presented in bar graphs were calculated by subtracting the last baseline value (before stimuli) to the highest fluorescence value after the stimuli.

### Mitochondrial H_2_O_2_ Analysis

HT22 cells were rinsed with Krebs medium and loaded with 10 μM mitochondrial peroxy yellow 1 (MitoPY1), a fluorescent probe that selectively accumulates in the mitochondria and reacts with H_2_O_2_. Cells were incubated with the probe during 20 min at 37°C in a humidified culture chamber containing 95% O_2_ and 5% CO_2_. Then, cells were rinsed with Krebs medium and fluorescence was read at 489 excitation and 540 emission in a Spectrofluorometer Gemini EM (Molecular Devices, United States).

### Analysis of Mitochondrial Oxygen Consumption Rate by Seahorse Analyzer

O_2_ consumption by HT22 cells, cultured as described before, was measured by using the XF24 flux analyzer according to Ferreira et al. (2018). HT22 cells were plated on XF24 microplates at a density of 0.3 × 10^4^ cells per well 48 h before the experiments. On the following day, cells were incubated with or without 1 μM AβO in the absence or in the presence of 1 μM boldine and cultured for an additional 24 h. The effect of boldine alone was also tested. On the day of experiments, cell culture medium was carefully removed and cells were washed two times with 1 mL DMEM5030 medium, pH 7.4, supplemented with 2 g/L glucose and 0.3 g/L glutamine to fully remove the previous medium. Then, the cells were incubated in 450 μL DMEM at 37°C in a CO_2_-free incubator for 1 h. Cellular bioenergetics was determined using the extracellular flux analyzer (Seahorse Bioscience). This system allows for real time, non-invasive measurements of oxygen consumption rate (OCR), which can be correlated with mitochondrial function/oxidative burst. Sequential injection of mitochondrial inhibitors oligomycin (2.5 μg/mL), FCCP (4 μM), and antimycin A (AntA; 4 μM) plus rotenone (2 μM) were added to evaluate basal respiratory capacity, maximal respiration in the presence of FCCP, oligomycin-sensitive O_2_ consumption coupled to ATP synthesis, proton leak, spare respiratory capacity and non-mitochondrial respiration as previously described (Ferreira et al., 2018). Results are expressed in picomoles of O_2_ per minute (pmol O_2_/min).

### Statistical Analysis

Data were analyzed by using Excel and GraphPad Prism 8 (GraphPad Software, San Diego, CA, United States) software and results are expressed as the mean ± SEM. Comparison among groups was performed using the Kruskal-Wallis test followed by uncorrected Dunn’s multiple comparisons test or one-way ANOVA followed by Tukey’s *post hoc* test. Statistical differences were represented as ^∗^*p* < 0.05, ^∗∗^*p* < 0.01, ^∗∗∗^*p* < 0.001.

## Results

### Boldine Interacts With Aβ_1__–__4__2_ *in silico*

**FIGURE 1:**
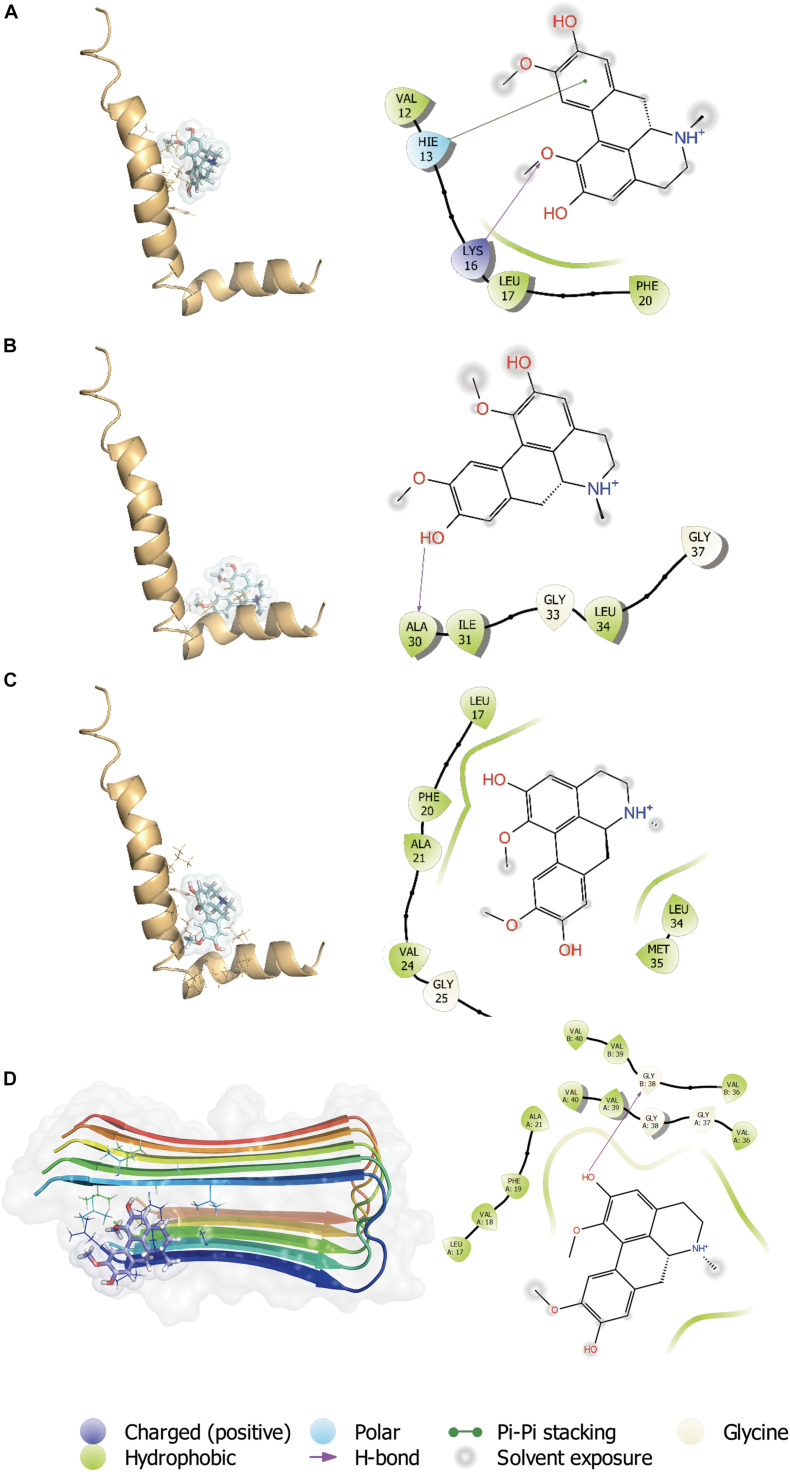
*In silico* interaction between boldine and Aβ_1__–__4__2_. Representative complexes of the interaction of Aβ_1__–__4__2_ (1IYT) with the **(A)** central region, **(B)** C-terminal, and **(C)** full peptide grids. Boldine also interacted with Aβ fibers (2BEG), with a higher docking score. **(D)** The insets show the ligand interaction diagrams (LIDs) with details of the Aβ_1__–__4__2_ residues involved in the interactions with boldine. The legend of the LIDs is shown at the bottom of the figure.

The C-terminal region of the Aβ peptide was found to play an important role in its aggregation, membrane association, pore formation and toxicity of the Aβ peptide (Peters et al., 2013). During a massive screening of small molecules with capacity to interact with the C-terminal region (Peters et al., 2020), we found several molecules having a high docking score and ΔG_*bind*_ (MM-GBSA), one of which was boldine (Figure 1A). Therefore, additional assessment of boldine and Aβ_1__–__4__2_ peptide (1IYT) docking interactions was performed considering 3 grids: central region (Figure 1B), C-terminal (Figure 1C) regions and full peptide (Figure 1D). For the interaction between boldine and the central region of Aβ (Figure 1B), a docking score of −2.047 was obtained. Here, boldine interacted with residues VAL 12, HIS 13, LYS 16, LEU 17, and PHE 20. A Pi-Pi stacking interaction was established with HIS 13 and a hydrogen bridge with LYS 16. For interaction of boldine and the C-terminal region (Figure 1C), a docking score of −1.427 was obtained and the data shows that boldine interacted with ALA 30, ILE 31, GLY 33, LEU 34, and GLY 37, establishing hydrogen bridges with ALA 30. For the interaction with the full structure (Figure 1D), a docking score of −3.373 was obtained and we found that boldine interacted with several residues, namely ALA 17, PHE 20, ALA 21, VAL 24, GLY 25, LYS 28, LEU 34, MET 35. Boldine also interacted with Aβ fibers (2BEG), with the higher score of −4.596. The residues found to interact with boldine were LEU 17, VAL 18, PHE 19, ALA 21, VAL 36 (x2), GLY 37, GLY 38 (2), VAL 39 (x2), and VAL 40 (x2). Therefore, boldine appears to affect several Aβ toxic species.

### Boldine Reduces Aβ Aggregation

**FIGURE 2:**
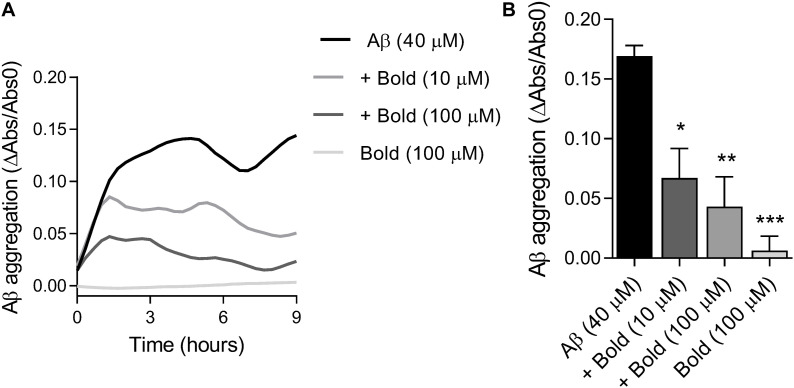
Effect of boldine on Aβ aggregation *in vitro*. **(A)** Representative traces of Aβ (40 μM) aggregation in the absence and presence of boldine (Bold; 10 or 100 μM) or boldine alone (100 μM). **(B)** Aβ aggregation levels after 9 h incubation (22–24°C) presented as the mean SEM of six replicates. Statistical analysis: One-way ANOVA followed by Tukey’s multiple comparison test; *p < 0.05, **p < 0.01, ***p < 0.001 when compared to Aβ.

Since we found that boldine was able to interact with Aβ, we further examined if this interaction was due to inhibition of peptide aggregation. Therefore, we assessed the aggregation of Aβ (40 μM) in the presence of two concentrations of boldine (10 and 100 μM) under constant agitation. Results in Figure 2A demonstrate that Aβ aggregation levels reached a plateau at about 4 h, that is similar to our recent study that reported the presence of AβO tetramers and nanometric structures using AFM (González-Sanmiguel et al., 2020). The levels of Aβ aggregation, as reflected by a reduction in turbidimetry, were measured up to 9 h of incubation and compared with boldine alone (incubated in the same conditions) (Figure 2A). Our results show that boldine inhibited Aβ aggregation in a concentration-dependent manner (*p* < 0.05 and *p* < 0.01 for 10 and 100 μM boldine, respectively) (Figure 2B). No signal was obtained in the presence of 100 μM boldine alone.

### Boldine Prevents Synaptic Impairment Induced by Aβ

**FIGURE 3:**
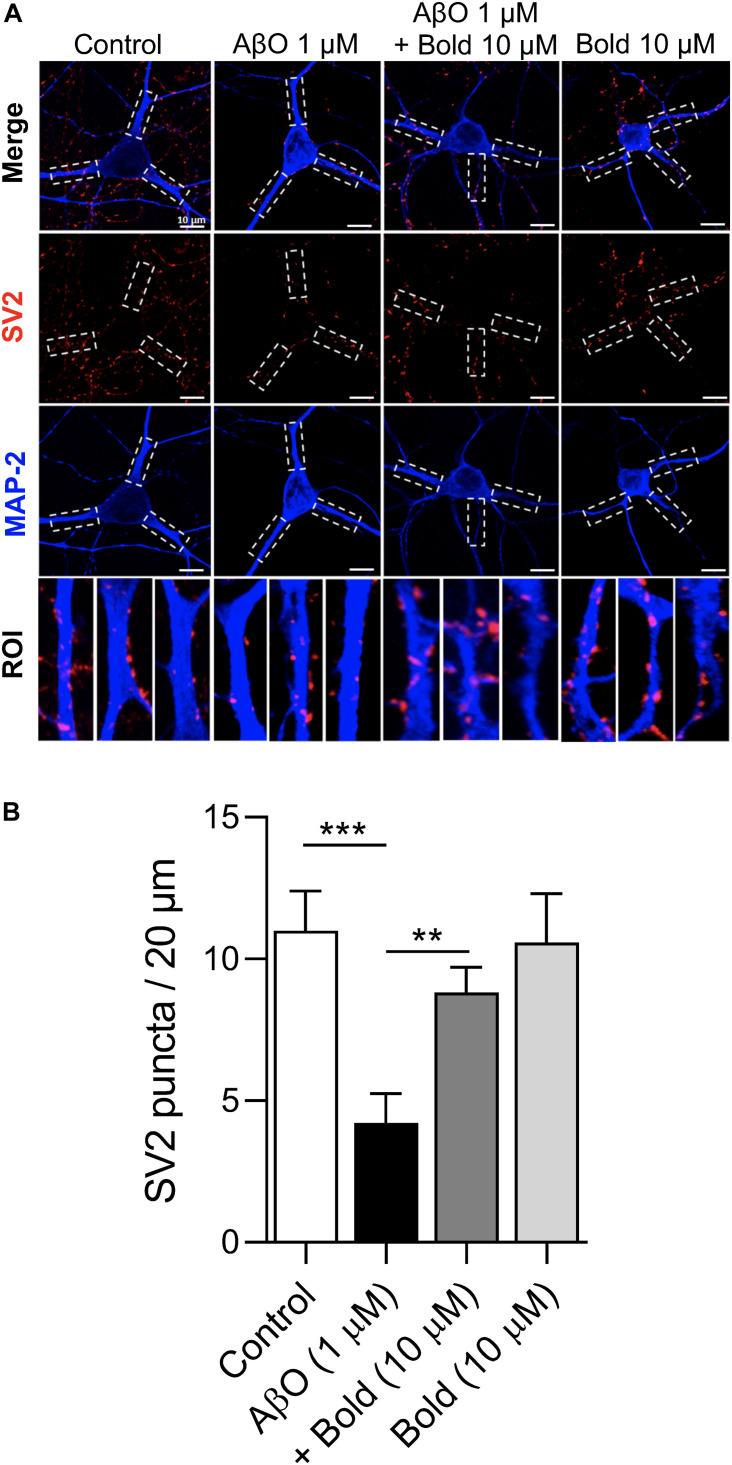
Effect of boldine on AβO-induced SV2 reduction. **(A**) Confocal micrographs showing SV2 immunoreactivity in hippocampal neurons (10 DIV) treated with 1 μM AβO in the presence or absence of 10 μM Boldine (Bold) for 48 h. Individual processes were identified as regions of interest (ROIs) (white segmented rectangles) for the determination of fluorescent puncta. Proximal neurites are measured from the soma up to 20 μm. **(B)** Quantification of SV2 puncta in the ROIs. Calibration bar is 20 μm. Results are represented as the mean ± SEM from at least 8 pyramidal shaped neurons (30 primary processes from three different coverslips for each condition). Statistical analysis: One-way Kruskal-Wallis ANOVA with Dunn’s *post hoc* test. ***p < 0.001 when compared to control and **p < 0.01 when compared to AβO.

To investigate if boldine was able to exert a protective action on the AβO induced synaptic impairment, similar to previous studies (Parodi et al., 2010; González-Sanmiguel et al., 2020; Peters et al., 2020), hippocampal neurons were treated for 48 h with 1 μM AβO in the presence of 10 μM boldine and further analyzed for SV2-labeled presynaptic vesicles by immunocytochemistry using confocal microscopy (Figure 3A). Quantification of SV2 puncta/20 μm process length showed a significant reduction in SV2 labeling in AβO-treated neurons (*p* < 0.05) and this effect was reduced when the neurons were co-incubated with AβO and 10 μM boldine (*p* < 0.05) (Figure 3B), suggesting a protective effect of boldine against AβO-induced synaptic damage. Moreover, incubation of the hippocampal neurons with 10 μM boldine alone had no effect on the SV2 puncta (Figures 3A,B).

**FIGURE 4:**
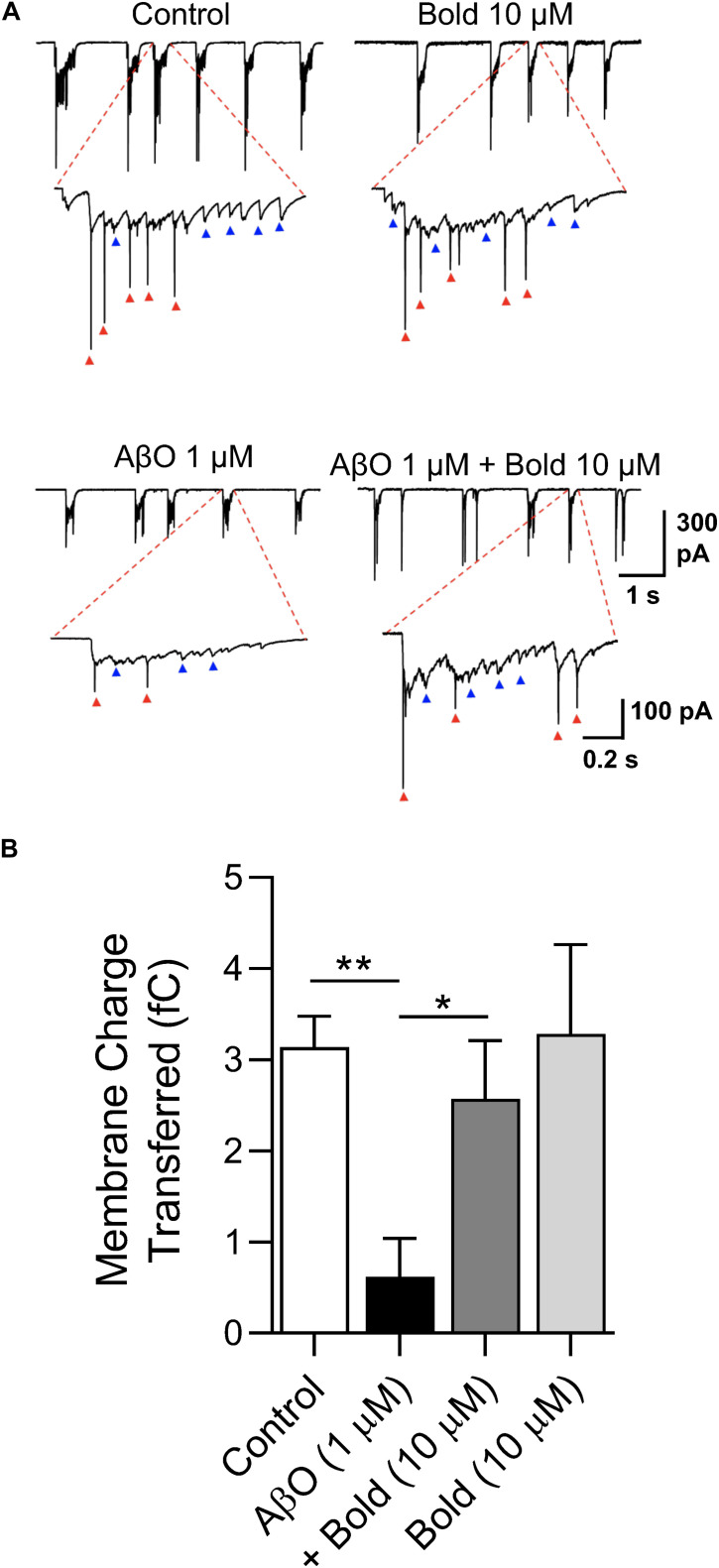
Characterization of postsynaptic currents in response to AβO treatment: Effect of boldine. **(A)** Whole-cell voltage-clamp recordings of post-synaptic currents obtained at a holding potential (V_*h*_) of –60 mV in hippocampal neurons (10 DIV) treated with 1 μM AβO in the absence or presence of 10 μM boldine (Bold) for 48 h. Blue arrows: bursts of synaptic currents; red arrows: spikes in current recording mode. **(B)** Quantification of charge transferred of post-synaptic currents. Results are represented as the mean ± SEM from at least 6 cells. Statistical analysis: One-way Kruskal-Wallis ANOVA with Dunn’s *post hoc* test. **p < 0.01 when compared to control and *p < 0.05 when compared to AβO.

To independently confirm the effect of boldine in recovering the SV2 protein labeling, and functionally preventing synaptic impairment, spontaneous synaptic currents were recorded at a holding potential (V_*h*_) of −60 mV in neurons treated with AβO in the absence or presence of boldine using the whole-cell technique. Under voltage clamp conditions, bursts of synaptic activity were recorded reflected by synaptic events (Figure 4A, blue arrows) and faster spikes (red arrows) (Figure 4A). All these events were integrated as charge transferred and quantified (Figure 4B). The data showed that, compared with the control conditions, 1 μM AβO caused a large reduction (*p* < 0.01) in the number and amplitude of the synaptic currents. Co-incubation of AβO with 10 μM boldine significantly recovered the presence of postsynaptic currents and synaptic transmission. Boldine alone had no effect on the functional activity.

### Boldine Prevents AβO-Induced Mitochondrial and ER-Ca^2+^ Accumulation

**FIGURE 5:**
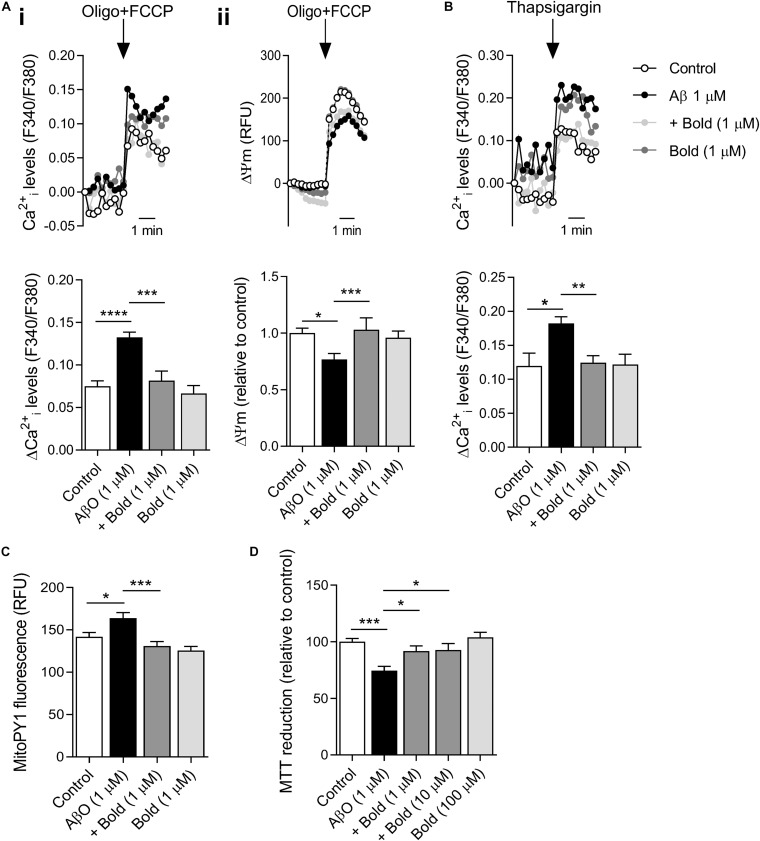
Effect of boldine on mitochondrial and ER-Ca^2+^ retention, ΔΨm, mitochondrial ROS levels and HT22 cell viability in AβO-treated HT22 cells. Cells were incubated with 1 μM Aβ in the absence or presence of 1 μM boldine and cultured for an additional 24 h. **(A)** Cells were co-loaded with Fura2-AM (5 μM) and TMRM^+^ (300 nM). Peak amplitude of Fura2 fluorescence ratio at 340/380 nm (i) and TMRM^+^ fluorescence at 590 nm (ii) following maximal mitochondrial depolarization induced by 2 μg/mL oligomycin plus 2 μM FCCP (Oligo + FCCP). Results are represented as the mean ± SEM of 3–5 independent experiments performed in quadruplicates. **(B)** Cells were incubated with Fura2-AM. Peak amplitude of Fura2 fluorescence ratio at 340/380 nm in response to 1 μM thapsigargin-induced ER-Ca^2+^ depletion (in Ca^2+^ -free medium). Results are represented as the mean ± SEM of 5 independent experiments performed in quadruplicates. **(C)** MitoPY1 fluorescence levels are represented as the mean ± SEM of 3 independent experiments performed in quadruplicates. **(D)** Cell viability was evaluated using the MTT assay and results are represented as the mean ± SEM of 3 independent experiments performed in quadruplicates. Statistical analysis: Kruskal-Wallis test followed by uncorrected Dunn’s multiple comparisons test; *p < 0.05; **p < 0.01; ***p < 0.001; and ****p < 0.0001 when compared to control or AβO.

AβO-induced mitochondrial and ER-Ca^2+^ overload plays a critical role on mitochondrial function, and consequently on bioenergetics and synaptic function (Reddy and Beal, 2008). Our results show that AβO-treated HT22 hippocampal-derived cells exhibited a significant increase in mitochondrial Ca^2+^ levels (*p* < 0.001), as evidenced by the increase in cytosolic Ca^2+^ rise following complete mitochondrial depolarization (with FCCP plus oligomycin) (Figure 5Ai). Moreover, AβO exposure enhanced ER-Ca^2+^ levels (*p* < 0.05) as observed after ER-Ca^2+^ depletion induced by 1 μM thapsigargin (selective SERCA inhibitor) in HT22 cells (Figure 5B). Boldine completely abolished the augmented mitochondrial and ER-Ca^2+^ retention induced by AβO (*p* < 0.001 and *p* < 0.01 for mitochondrial and ER-Ca^2+^, respectively) (Figures 5Ai,B). Nevertheless, boldine itself had no effect on Ca^2+^ accumulation in either organelle (Figures 5Ai,B). These results suggest that boldine is able to alleviate Ca^2+^-mediated ER and mitochondrial dysfunction caused by AβO.

### Boldine Interferes With AβO-Mediated Changes in Mitochondrial Function and ROS Levels, Alleviating Cell Viability

To further assess the role of boldine on mitochondrial function, ΔΨm was assessed using TMRM^+^, a fluorescent probe that predominantly accumulates in polarized mitochondria. Our results showed that AβO-treated HT22 cells exhibited a significant decrease in ΔΨm (*p* < 0.05), which was largely prevented by 1 μM boldine (*p* < 0.001) (Figure 5Aii). Treatment with boldine alone did not exert any effect on ΔΨm (Figure 5Aii). These data highly suggest that compromised mitochondrial function in AβO-treated HT22 cells can be restored by boldine.

Based on these changes, we also examined mitochondrial ROS in HT22 cells labeled with MitoPY1, a probe that selectively reacts with H_2_O_2_ produced by mitochondria. Our results indicate that Aβ enhanced mitochondrial ROS levels (*p* < 0.05) and this effect was largely prevented by co-treatment with boldine (*p* < 0.001) (Figure 5C). Boldine had no effect on mitochondrial ROS production in control cells (Figure 5C). HT22 neural cells incubated with 1 μM AβO for 24 h also showed a decrease in cell viability as evaluated by the MTT assay. According with the previous results, boldine (1–10 μM) had a neuroprotective role in this AD cell model (*p* < 0.05) (Figure 5D). Data shows that even at a high concentration (100 μM) boldine does not affect cell viability (Figure 5D).

**FIGURE 6:**
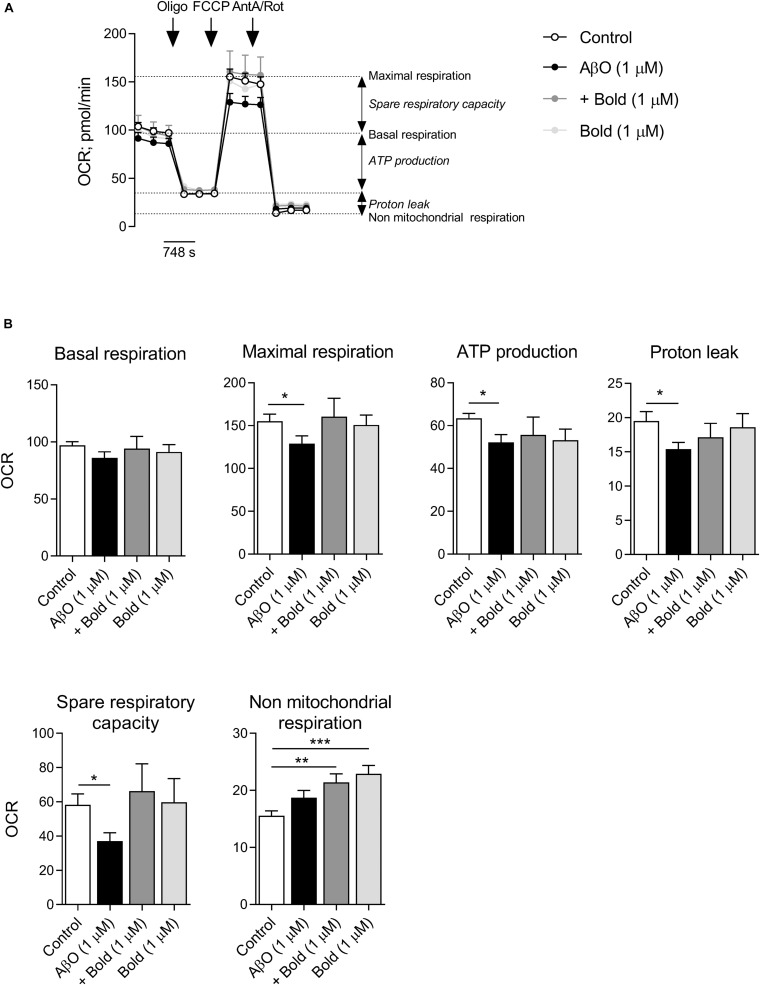
Effect of boldine on oxygen consumption rate (OCR) in AβO-treated HT22 cells. Cells were incubated with 1 μM Aβ in the absence or presence of 1 μM boldine and cultured for an additional 24 h. **(A)** Traces of mitochondrial respiration before and after oligomycin (oligo), FCCP and antimycin A (AntA) plus rotenone (Rot) injection in cells treated with 1 μM Aβ and 1 μM boldine for 24 h. **(B)** Basal and maximal respiration, ATP production, proton leak, spare respiratory capacity and non-mitochondrial respiration. Results are presented as the mean ± SEM of 3–5 independent experiments performed in duplicates. Statistical analysis: Kruskal-Wallis test followed by uncorrected Dunn’s multiple comparisons test; *p < 0.05; **p < 0.01; and ***p < 0.001 when compared to control.

Because regulation of mitochondrial Ca^2+^ levels is relevant for mitochondrial overall activity, we analyzed mitochondrial respiration profile and ATP synthesis in cells exposed to AβO and/or boldine. Mitochondrial respiration was analyzed in HT22 cells incubated with AβO in the absence or presence of boldine (1 μM) by using a Seahorse XF24 flux analyzer (Figure 6) in which basal respiratory capacity, maximal respiration (in the presence of FCCP), oligomycin-sensitive oxygen consumption coupled to ATP synthesis, H^+^ leak, spare respiratory capacity and non-mitochondrial respiration were measured (Figures 6A,B). Our results clearly demonstrated significant decreases in maximal respiration, ATP synthesis, H^+^ leak and spare respiratory capacity in AβO-treated HT22 cells, while in the presence of AβO plus boldine oxygen consumption rates were similar to control conditions when assessing maximal respiration, H^+^ leak and spare respiratory capacity (Figures 6A,B). AβO or boldine induced no significant changes in basal respiration, but boldine *per se* enhanced non-mitochondrial respiration (Figures 6A,B). No changes in glycolytic activity were detected under these conditions (data not shown).

These data demonstrate that cytoprotective effects of boldine in cells exposed to AβO largely involve its capacity to ameliorate mitochondrial function and redox profile.

## Discussion

The present study shows that boldine can interact with Aβ *in silico* and inhibit its aggregation at low micromolar concentrations. Boldine was also able to attenuate the synaptic failure induced by AβO, along with calcium and mitochondrial dyshomeostasis, suggesting that it could act as a natural anti-AD compound.

The analysis of *in silico* docking between boldine and Aβ provided an initial evidence supporting their association. This positive identification resulted from performing a much larger screening, looking for small molecules that could interact with the C-terminal region of Aβ. Previously, using FRET, aggregation assays and other techniques, we found that a small pentapeptide having the sequence of Aβ was able to associate and effectively block its synaptic toxicity (Peters et al., 2013). More recent studies using *in silico* docking, molecular dynamics, and *in vitro* and *in vivo* approaches allowed us to identify a small molecule with similar protective actions on the peptide (Peters et al., 2020). The present docking study expands on these previous results and shows that boldine, a Chilean tree-derived natural compound, interacts with several amino acids along the Aβ sequence such as glycine 33 and 37, previously shown to be important for the Aβ association to artificial lipid bilayers (Lin et al., 2008) and self-aggregation, in agreement with the idea that boldine can interfere with the formation of higher order complex species like oligomers and fibrils. We also showed that boldine interacted with glycine 25, which belongs to a motif of 14 residues (i.e., GLY25-GLY37) important for its aggregating properties (Jarrett and Lansbury, 1993; Peters et al., 2013). Boldine also interacts with histidines 13 and 14, recognized to be important for the permeation of ions through the amyloid channel (Nakamura et al., 2007). Thus, the data provide *in silico* evidence for an interaction between Aβ monomers and boldine. Interestingly, Arispe (2004) and Arispe et al. (2008) previously reported that small molecules that form complexes with these histidines blocked both the channel opening and its cytotoxicity. Consistent with an interaction between Aβ and boldine, the alkaloid was effective inhibiting the aggregation of Aβ *in vitro*. Interestingly, apomorphine, another structurally related aporphinoid, also showed anti-aggregation properties suggested to be related to structural changes of the peptide when interacting with apomorphine (Hanaki et al., 2018). As an anti-aggregation agent, apomorphine was able to inhibit the formation of amyloid plaques in the brain of 3xTg-AD mice (Himeno et al., 2011). At this moment, however, we do not have additional information on particular Aβ species that are more affected by boldine. It is interesting to note that apomorphine and boldine are aporphine alkaloids, thus they share a number of chemical and biological properties. In this way, and similar to apomorphine, boldine inhibited Aβ aggregation at very similar concentrations. A recent study using nuclear magnetic resonance showed that apomorphine interacted with Arg5, His13,14, Gln15, and Lys16 of the Aβ_*1–42*_ monomer (Hanaki et al., 2018). Interestingly, our results show that several of these residues also participate in boldine interactions. Finally, the present *in silico* analysis also show that Aβ fibers can interact with boldine, suggesting that it can actually affect several species. Therefore, the inhibitory capacity of boldine on Aβ aggregation can be attributed to its interaction with the C-terminal region of the peptide, potentially interfering with the interaction of the peptide with the cellular membrane (Kim and Hecht, 2006; Lin et al., 2008). Interestingly, the study of macroscopic amyloid burden in 2xTgAD mice was not modified by long-term treatment with boldine added to drinking water (Yi et al., 2017). This negative result could be explained because the experiment involved a somewhat late stage of plaque formation (6 months) and because addition of boldine to drinking water does not guarantee brain delivery at therapeutic concentrations.

The synaptotoxicity produced by AβO is likely initiated by its capacity to alter membrane permeability causing a large, unregulated increase in intracellular Ca^2+^ levels through a cation-conducting pore at the plasma membrane. This leads to an early enhancement in neurotransmission that is followed by synaptic vesicle depletion and a loss of intercellular communication (Sepúlveda et al., 2014). The synaptotoxicity of AβO may be also related to the alteration of excitatory ligand-gated ion channels, such as the NMDARs and/or functional alterations of intracellular ion stores (e.g., ER, mitochondria), promoting Ca^2+^ dyshomeostasis (Shankar et al., 2007; Ferreira et al., 2012b). Results obtained by analyzing SV2-labeled presynaptic puncta, a good presynaptic marker (Parodi et al., 2010), indicated that boldine protects against AβO-induced synaptic impairment. The recovery in the presynaptic marker with boldine was in agreement with the increase in functional synaptic transmission found in AβO treated neurons. In previous studies, also supporting beneficial synaptic effects of boldine, it was found that a dose range of 3–25 mg/kg boldine administered to scopolamine-treated mice improved memory function (Dhingra and Soni, 2018) and ameliorated brain damage and cognitive deficits after cerebral artery occlusion (De Lima et al., 2016).

By altering Aβ aggregation, boldine can prevent AβO-induced Ca^2+^ accumulation in the cytosol. As such, AβO-induced Ca^2+^ accumulation in ER and mitochondria was alleviated following boldine treatment, and mitochondrial function was improved. ER and mitochondria contact sites establish microdomains for inter-organellar Ca^2+^ signaling at MAMs, which contain, efficient Ca^2+^-transport systems. Thus, boldine interfered with AβO-mediated direct or indirect mitochondrial effects linked to ATP production (e.g., complex IV) and oxidative stress (Rhein et al., 2009; Beck et al., 2016). In this respect, boldine alleviated the reduction in maximal respiration and largely increased spare respiratory capacity in cells treated with AβO, thus potentially enhancing ATP generation by oxidative phosphorylation in case of a sudden increase in energy demand.

Concordantly with changes in respiratory reserve, boldine interfered with AβO-mediated changes in ΔΨm and ROS levels. Previously, treatment with boldine (10–20 mg/kg) was shown to restore ΔΨm and ROS levels in a murine model of renal dysfunction (Heidari et al., 2019). In addition, boldine restored ΔΨm in high dopamine stressed PC12 cells (Youn et al., 2002). Under AβO conditions, voltage-dependent anion channel protein (VDAC) is overexpressed and aggregated inducing a decrease in ΔΨm (Smilansky et al., 2015). Indeed, small molecules can inhibit VDAC aggregation, positively regulating ΔΨm and energy imbalance caused by Aβ (Ben-Hail et al., 2016). Acting as a small molecule in AβO-treated hippocampal cells, boldine may have a similar effect to restore ΔΨm. Therefore, control of mitochondrial Ca^2+^ signals critically regulate mitochondrial activity and maintain the ΔΨm, thereby affecting cell metabolism and survival.

In addition, boldine prevented mitochondrial H_2_O_2_ levels induced by AβO, which is in agreement with previous studies in several models indicating that the neuroprotective effects of boldine are due to its antioxidant effects (Konrath et al., 2008; De Lima et al., 2016). Boldine (50 mg/kg) inhibited ROS production and lipid peroxidation in hypertensive and diabetic rats (Jang et al., 2000; Hernández-Salinas et al., 2013; De Lima et al., 2016; Gómez and Velarde, 2018). In addition, Youn et al. (2002) reported that boldine inhibited the opening of the mitochondrial membrane permeability transition pore (mPTP) that leads to an increase in ROS. Dhingra and Soni (2018) also found that boldine (6 mg/kg) reduced malondialdehyde (MDA) levels, a product of lipid peroxidation and an indicator or oxidative damage. This is in agreement with the well described antioxidant activity of boldine, which can react with free radicals due to its polyphenolic structure (Valenzuela et al., 1991).

**FIGURE 7:**
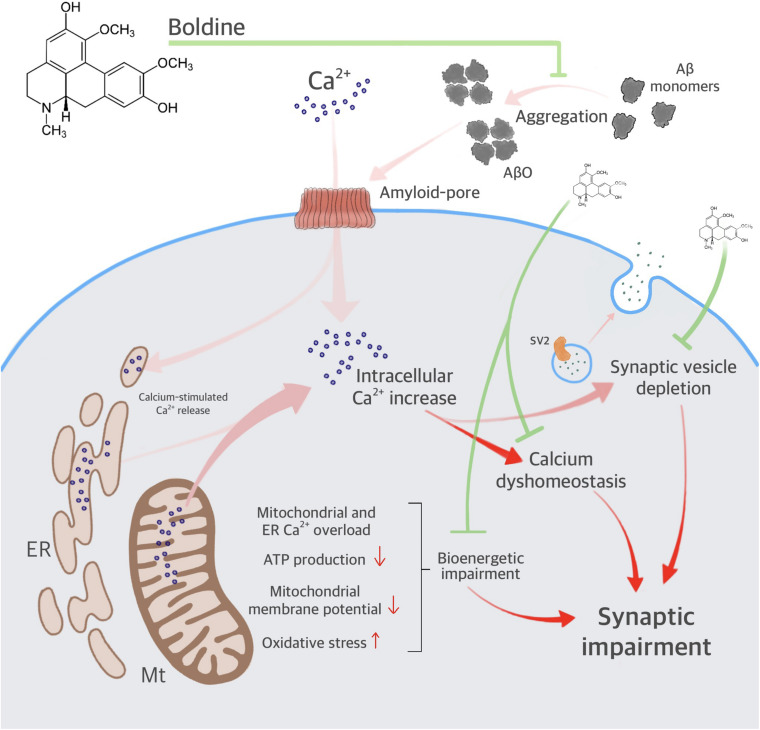
Proposed mechanisms for boldine neuroprotection against AβO-induced synaptic failure and mitochondrial dysfunction. The scheme depicts AβO aggregation, increasing intracellular Ca^2+^, and synaptic impairment. AβO also increases endoplasmic reticulum (ER) and mitochondrial (Mt) Ca^2+^ accumulation, contributing to calcium dyshomeostasis and bioenergetic impairment. Deregulation in intracellular Ca^2+^ leads to synaptic failure. Boldine interferes with the toxicity of AβO by inhibiting major steps in the amyloid cascade includying membrane and organelle dyshomeostasis.

Our data indicate that boldine interacts with the Aβ_1__–__4__2_ peptide, reduces AβO aggregation, and attenuates AβO-induced synaptic impairment (Figure 7). Furthermore, boldine restores AβO compromised mitochondrial function, including ER- and mitochondrial Ca^2+^ accumulation, disruption of ΔΨm, overproduction of mitochondrial ROS and mitochondrial respiration, namely maximal respiration, proton leak, and spare respiratory capacity in AβO-treated cells (unchanged OCR values when compared to the control). Therefore, the neuroprotective effect of boldine may result from: (1) Interaction with residues implicated in the association of Aβ to the membrane and the formation of channels permeable to Ca^2+^; (2) Interaction with residues implicated in ion permeation (mainly Ca^2+^) through the cellular membrane (i.e., histidines 13 and 14); (3) Direct interaction with Aβ, preventing ER and mitochondria deregulation due to Ca^2+^ rise, thus ameliorating mitochondrial function. In conclusion, low concentrations of boldine interfere with Aβ aggregation, prevent synaptic failure and organelle dysfunction, centering on mitochondria as one of the relevant players relevant for cell survival, and thus constituting a potential neuroprotective drug in AD.

## Data Availability Statement

The raw data supporting the conclusions of this article will be made available by the authors, without undue reservation.

## Ethics Statement

The animal study was reviewed and approved by the Ethics Committee at the University of Concepcion.

## Author Contributions

JT, EF-P, IF, ACR, and LA wrote the manuscript, designed *in vitro* experiments, and discussed the results. CB designed and performed *in silico* studies and analysis. JT, EF-P, NR-L, BP-C, LP-P, and DM carried out material preparation, data collection and analysis of *in vitro* studies. All authors read and approved the final manuscript.

## Conflict of Interest

The authors declare that the research was conducted in the absence of any commercial or financial relationships that could be construed as a potential conflict of interest.
